# Research and the Medical Student

**Published:** 1920-09-04

**Authors:** 


### RESEARCH AND THE MEDICAL STUDENT.

Since the last educational numbers appeared so much of the then predicted resettlement in our medical schools has begun to occur, and the principles thereof have been under such frequent and searching discussion, that it is now possible to review the situation with a feeling of far greater certainty. The future of medical practice in civil life and in the Services is dealt with exhaustively in other pages of our present issue, and in this short article we will devote our attention entirely to a very prevalent idea held by the medical students now entering or about to enter the profession. Again and again one hears the parents of the academically clever youth, and, indeed, the youth himself explain that the ideal which draws him to medicine is that of the glories and achievement of research.

Now, in the great majority of instances, the conception of the modern medical situation which lies at the root of this decision is that there is a hard-and-fast dividing line between the ordinary routine of private or institutional practice and Research, as spelt with the big R. Fundamentally this may be correct; but actually it applies only to the extreme instances. On the one hand we have medical practice regardlessly driven as a commercial enterprise, on the other we have the worker who is entirely occupied in investigational work and entirely disinterested of financial considerations. Between the two are to be found every possible grade, mixtures in every possible proportion of two outstanding professional types. In brief the modern tendency is to expose the deficiencies of the former and to free the latter's work from the atmosphere of the water-tight compartment. Both types must receive such training as will produce sympathetic mutual understanding, and ultimately the average practitioner must come more seriously to act upon the true principles of medical investigation, and the research worker to realise more fully the real application of his work to practical, everyday medicine. All the recent discussions on the "beginnings of disease," preventive medicine, the revised medical curriculum, etc., have tended in this same direction.

### Forecasting a Career.

The student therefore who is now forecasting his career and taking as his type and the direction of his ambition one or more of the already established professional men around him must realise that his training will be inevitably and essentially different from theirs. It will be more comprehensive, far fewer academic facts will be learnt simply to be forgotten, and every attempt will be made by his teachers to provide him with a correctly proportioned picture of the possible medical fields before him. Also they, far better than his parents, can correctly judge into which channel his own individuality and relative skill will most advisedly take him. It is impossible here even to outline the available paths now open, but the point we would make is that it is only after the first half of the medical curriculum has been passed that it is advisable for either the student or his preceptors definitely to formulate his professional ambitions.

Coming, then, to the particular subject of investigational medicine, every student must remember that if he is nowadays even approximately to live up to the ideals of his profession he must, whatever practice he takes up, keep the true spirit of research well to the forefront of his mind. But if, on the other hand, he definitely decides to seek fame in the realms of research as such, then he must not only expend more time, money and trouble over his preliminary training, but he must also be possessed of special mental ability, a fact upon which he and his teachers will advisedly decide as he goes through his medical school. If we are dealing with true research, we must, in the main, discard the financial consideration. Great efforts are being made, and they are at least partially successful, in raising endowments and general monetary support for this urgently necessary medical work; but there is no doubt that those responsible for the financial side of the matter will, in the end, take great care that only the best men are thus sheltered in their efforts, and competition will be correspondingly keen among the younger generation who would enter this sphere. We need the best men for it, and, quite justifiably, only the best will be selected.

### Attractive and Valuable Fields.

Nevertheless there is no doubt that of all sciences, medicine offers some of the most attractive and economically valuable fields in which investigational work may be carried on, and the war-named principle of team work is certain to be the key-note of all future endeavours. The Medical Research Council, as one famous example, is doing everything possible to foster this method, and, following back their already published records, the young man should at once realise that, to take his part successfully in such a team, he must give himself, beyond a thoroughly comprehensive general training, an expert facility in one of the many specialised branches he sees exemplified around him.

One of the points of greatest importance to the research student is the type of teaching which is provided by the school he selects. In short, the old school, good as it was, will not serve for the future. As Dr. Arbour Stephens, in a recent communication, says, dogmatism may be necessary to some extent in the first stages of student life, but when it tends to be combined with the doctrine of infallibility, the result is bad insomuch as it crushes out of existence all attempts at research thinking on the part of the ordinary student. For progress it is absolutely necessary that individual thinking and the spirit for research should be cultivated amongst those who have to go out to the wilds as well as those who indulge in such luxuries simply as an aid to rise to the heights of professional success in Harley Street.

